# Notes from Practice—Obstruction of the Bowels, with History and Autopsy of Three Cases*Read before the Buffalo Medical Club.

**Published:** 1882-02

**Authors:** Thomas Lothrup


### Notes from Practice—Obstruction of the Bowels, with History and Autopsy of Three Cases.*

Reported by Thomas Lothrup, M. D.

The term "Obstruction of the Bowels" comprehends a wide range of seemingly diverse diseases, such as formation of membranous bands, rotation of the intestine upon its own axis, compression from tumors, enlarged ovaries, intussusception, stricture, foreign bodies, paralysis, spasm, etc., all of which tend, under favorable circumstances and conditions, to one result, obstruction. While not aiming to present anything original, the history and progress of three interesting cases of this very serious and often fatal disease, in which the autopsy revealed intestinal lesions, which it was difficult to positively diagnose, especially in its early stages, and for which neither surgical nor medicinal measures afforded relief, may be suggestive and indeed profitable.

The first case occurred in July, 1867. The patient, Mrs. B., aged 40 years, had been the subject for many years of that most common ailment among women, constipation, and for its relief had resorted to laxatives, at first selecting the milder remedies of this class, and, gradually, as the intestines became accustomed to their use, using the stronger cathartics. For two weeks previous to my attendance, patient was under the treatment of a well-known botanic physician of this city, whose remedies failed to afford any

* Read before the Buffalo Medical Club.

relief from present suffering, nor any hope from the impending fate awaiting her. In closely interrogating the patient, it was found that for many months the fæces had been less in quantity and greatly diminished in size, as if the tube, through which the intestinal contents passed, diminished in diameter, had elongated the fæces in their passage; cathartics also aggravating the obstruction they were administered to overcome. The patient was suffering from intense pain to the left and below the umbilicus; the abdomen was tender to the touch and tympanitic; there was nausea, vomiting and great depression of the vital powers. Calling in consultation the late Dr. Sanford Eastman, the seat of the obstruction was located at or near the sigmoid flexure of the colon, and the introduction of the rectum-tube and the injection of large quantities of warm water recommended, with the internal administration of opiates to relieve pain. The patient gradually sank and died three weeks after presenting positive symptoms of intestinal obstruction.

The autopsy confirmed the diagnosis, the obstruction being at or about the middle portion of the flexure, and was caused by a fibrous constriction, making the canal impervious. The colon above the constriction was filled with hard fæcal masses, evidently the accumulation of many weeks or months, while the intestinal walls presented the usual dark-red hue.

The second case occurred in October, 1867, at the Erie Co. Penitentiary, while I was attending physician to that institution. The patient, aged 45, a native or Ireland, like the most of those sent to penal and reformatory institutions, had depreciated his vital forces by prolonged dissipation. The usual symptoms of pain, tenderness, tympanitic abdomen, nausea, vomiting, etc., were present. But little could be learned of the previous history of the case. As soon as I became satisfied of the nature of the disease, I summoned Dr. Rochester, who confirmed the diagnosis of obstruction of the bowels at or near the sigmoid flexure, advised the continuance of opiates, large enemata, the warm-bath until symptoms of relaxation were manifest. The rectum tube was also used in this case, but our efforts to pass it through the sigmoid flexure were unavailing, the injections passing away almost as they were thrown up. The patient lived about ten days after admission.

The post-mortem examination revealed extensive disease of the entire sigmoid flexure, occlusion of the intestinal canal at that point, thickening of the intestinal walls with hard deposits in the mesentery and between and involving the intestinal coat. The disease was evidently scirrhus. Many interesting points in this case I failed to obtain, and much of its value in a professional point of view is lost from the impossibility to learn anything positive of his former history and previous symptoms. The importance of the case in this connection rests in the cause of the obstruction, scirrhus, and the failure or impossibility to accurately diagnose its precise nature.

The third case is of more recent date. Wednesday, April 12, 5 P. M., was called to Fred. A. G., aged 20 years. Symptoms: pulse 96; tongue slightly coated; nausea; vomiting; palor around the mouth; pain in umbilical region, with the usual exacerbations and remissions, peculiar to colic. Inquiry as to diet disclosed the indiscretion of his having eaten a quantity of orange peel before breakfast the previous morning. The day before the patient had attended to his ordinary duties, not manifesting the usual mental vivacity and physical activity peculiar to him. With these symptoms and such other data as could be gathered, I diagnosed colic from indigestion; gave ¼ gr. morphia subcutaneously, and waiting one-half hour, during which relief from pain was experienced. I order morpia ¼ gr., to be administered by the stomach during the night, as often as was necessary to subdue pain, with hot fomentations over abdomen, etc., etc.

Thursday, 12 M. Pulse 70; pain almost entirely absent; no tenderness of abdomen or tympanitis; nausea and vomiting subsided. Diagnosis of the day previous confirmed; prognosis favorable. Ordered a cathartic of Seidlitz pd. the following morning, in case the bowels failed to be moved. At about 4

o'clock, P. M., the pain returned with greater severity, the abdomen became tender, and tympanitis, nausea, and vomiting returned; the palor around the mouth, which was not noticable at noon, became more marked, with indeed all the severe symptoms of obstruction of the bowels. Ordered morphia, hypodermically, and per stomach, with hot fomentations. The patient passed a comfortable night under the influence of full doses of the opiate.

Friday, 9 A. M. Pulse, 108; temperature, 99°; pain not severe; tympanitis not excessive; nausea constant, and frequent vomiting, and at irregular intervals; no action of the bowels. The injections followed by increased nausea and vomiting, but morphia, ¼ gr. internally, to be again tried, repeated ad lib.

Friday, 10 P. M. No improvement; pulse, 106; temperature, 99; morphia continued; hot fomentations; enemata of warm water, with castor oil and turpentine.

Saturday, A. M., 7 o'clock. Pulse 130, with marked depression of the vital powers; stercoraceous matter ejected from stomach during the night, in increasing quantities as morning approached. I immediately threw into the rectum one gallon of warm water, and by means of a compress, firmly held to the anus, retained the fluid until 8.50 when it was ejected, somewhat colored with fæcal matter. At 9 o'clock the same quantity of warm water was repeated with 2 oz. whiskey and 1 oz. of ol. tereb., which was soon evacuated without trace or odor of fæces. Calling Drs. Wetmore and Harrington in consultation, it was decided to continue morphia hypodermically and per stomach with champagne. At 3.40 P. M., patient died. Autopsy 24 hours after death; liver, kidneys, stomach, healthy; entire colon normal. The small intestines natural until the ilium was reached, when the dark-red color, peculiar to obstruction, was detected, and at a point 10 inches from the ileo-cœcal valve, a constriction of the intestine was found, perfectly occluding the canal, and the gut invaginated for eight inches, from the point of constriction. A close examination revealed the fatal nature of the obstruction and the impossibility of rendering more than temporary relief.

These cases reveal three distinct types of obstruction. The first was evidently the result of constipation of long continuance, which, with the accumulation of fæcal matter and the influence of drastic cathartics, produced the fatal constriction.

The case is not without interest, in view of the frequency with which attention is directed to constipation, especially in women of nervous temperament, among whom the practice of drugging is resorted to, in preference to the more philosophical or rational adoption of hygienic and dietetic measures. The sigmoid flexure being the more contracted and tortuous portion of the colon is the principal seat of obstruction from this cause. The differential diagnosis is facilitated by the accumulation above the point of constriction, detected through the abdominal walls, and the failure to force the injection of water above the point, and also its rapid evacuation.

The second case from *scirrhus* of the sigmoid flexure, I am unable to obtain a full and complete history upon which to base an opinion, as to the period of its existence. The patient complained of symptoms on his admission, which in a short time assumed the alarming character—peculiar to permanent obstruction—and the case progressed rapidly to a fatal issue, only the autopsy revealing the exact nature and location of the disease.

The third case from intussusception of the ilium is one of rare interest, as much on account of the rapidity of its course as from the subsidence of all active symptoms the day following my first visit. In closely interrogating the family in regard to the habits of the young man previous to the attack, a clue was obtained to the real cause of the invagination, and a rational explanation afforded of its inception. In the rear of his residence, in excavating the gravel, a ditch or canal had been formed from 12 to 15 feet in width, with an embankment on the distal side. On monday evening, April 10th, Mr. G., for the purpose of exercise, made several attempts to jump to the opposite embankment; and the violent effort of running the distance of two or three rods in order to acquire the impetus necessary to perform the feat, while the leap, with the sudden concussion following, evidently produced the invagination of the intestine of which he died. This explanation is strengthened by the want of his usual sprightfulness of mind and body observed by his relatives on the day following the exercise, although he made no complaint of pain on that day. The case also presents another feature of interest in the rapidity of its course, only three days and six hours intervening between the time of the first complaint of pain and the fatal issue, in striking contrast to cases No. 1 and 2, in which death occurred after many weeks.

A few words as to the general and local treatment of obstruction will appropriately supplement the history and autopsy of the cases which constitute the basis of this paper. The principal indication is the relief of pain. Opium and its alkaloids meet this requirement, and they should be used freely, the limit of their administration being reached when the abdominal pain, of which the patient mostly complains, is so far overcome that rest, general and local, is secured. Special emphasis may be given to this principle in the therapeutics of all enteric diseases of an inflammatory type, and special dependence may be placed upon opium, both as an anodyne and as an antiphlogistic. With the same emphasis we would warn against the use of cathartics, which may be one of the factors in the causation of the obstruction as in the first case, cited above.

Reaching the obstruction through the rectum is often the only avenue for the introduction of remedies. In case the seat of the obstruction is in the small intestines, medication per rectum is not as efficacious as when located in any portion of the large intestines, and for very obvious reasons. But even here it is important to make the attempt by introducing the rectum tube as far up the colon as possible. The difficulty in passing the sigmoid flexure cannot always be overcome, but having carried it as high as possible, the gradual and slow injection of warm water, even to the amount of a gallon or two gallons until the canal will hold no more, will in some cases, prove efficacious.

These injections should be repeated at intervals, adding whisky in case the powers of life give indications of failure. Dr. Watson mentions a successful case from the inflation of the gut with air, by means of bellows, two hours after which the patient passed a natural fæcal stool and recovered. The use of the induced and constant current has been followed with success in cases ot obstruction, due to impacted fæces. The injection of a quart of cold ice-water has proved successful. Inverting the body by taking the patient by the feet over the shoulders of an attendant has been resorted to.

Tobacco enemeta are advised, but in their use, extreme caution should be observed.

After these measures have failed our only resort is to gastrotomy or enterotomy, and the formation of an artificial anus.

It is plain that the insuperable obstacle in the way of success in enterotomy is the uncertainty of the real cause of the obstruction and of its precise location.

Taking either of the three cases, recorded in this paper, as a fair test of the feasibility of the operation and viewed in the light thrown upon them by the autopsy, the operation would have been a useless expenditure of time and labor. That cases may be presented in which, if the operation be performed in time, success may be attained, cannot be doubted, but as a general rule the operation is delayed until the vital powers have become well-high exhausted, and it is then suggested as a denier resort. The delay is fatal to success in the majority of cases, and the late hour at which it is performed, destroys almost the last hope for the patient. But in case after carefully weighing all the symptoms, especially the vital forces of the patient, it is deemed proper to make the effort to overcome the obstruction by cutting through the abdominal parietes, how shall it be done? For gastrotomy, the simply crucial incision, the longitudinal commencing just below the ensiform cartilage, and extending three inches downward, and the transverse extending from the middle of the longitudinal, one and an half inches on either side; carefully seizing the stomach, the organ is drawn forward and stitched with silver wire to each limb of the cutaneous flap, after which an opening is made into the organ, and the cause of the obstruction removed.

For enterotomy, the operation is performed at or near the seat of obstruction, if it can be definitely determined, by making an incision of adequate length in the direction of the muscular fibres—and an examination of the intestine made. If the construction is firm, the only resource is an artificial anus, and all the subsequent inconveniences resulting therefrom in case the patient survives.

Surveying the intestinal track, its structure and important functions in the animal economy, surprise may be excited, not at the infrequent occurrence of fatal organic lesions of this important viscus, but in view of its extensive surface or area, that so few serious obstructions should come to our notice. As the principal avenue through which the body is nourished, and also from which the excrementitious matter is carried away, the alimentary canal assumes an importance, inferior to none other, in its relations to the living organism, both in health and disease. It bears the most severe strain upon its functions through unnatural appetites and artificial aliments, but resents with fatal effect any interference with the integrity of its structures or the proper performance of its duties.

This paper has been purposely confined to practical considerations connected with the subject under discussion. While aiming to draw rational deductions from the cases presented, the object in view has been to make the path clear to those of the Club who may have to combat one of the most unpleasant and fatal of the morbid conditions, which in future may come under your professional observation.

